# Pylorus-preserving pancreatoduodenectomy preserving blood supply for pancreatic cancer with a history of proximal gastrectomy and sigmoidectomy: a case report

**DOI:** 10.1186/s40792-024-02063-w

**Published:** 2024-11-21

**Authors:** Yuto Nakane, Takayuki Minami, Yasuhiro Kurumiya, Keisuke Mizuno, Ei Sekoguchi, Gen Sugawara, Masaya Inoue, Takehiro Kato, Naohiro Akita

**Affiliations:** Department of Surgery, Toyota Kosei Hospital, 500-1 Josui-cho, Toyota, Aichi 470-0396 Japan

**Keywords:** Pancreatoduodenectomy, Right gastroepiploic vessel, Proximal gastrectomy, Sigmoidectomy, SMA stent

## Abstract

**Background:**

Blood supply to the remnant stomach should be preserved during pancreatectomy in patients with a history of gastrectomy. Moreover, ischemic complications should be considered when performing pancreatoduodenectomy in patients with celiac axis and superior mesenteric artery (SMA) stenosis. However, whether these surgical procedures can be safely performed remains unclear.

**Case presentation:**

A 75-year-old man had a history of laparoscopic proximal gastrectomy (PG) with double-flap technique for gastric cancer and laparoscopic sigmoidectomy for sigmoid cancer treated 4 years ago. Follow-up computed tomography (CT) revealed an irregular nodular tumor measuring 13 mm in the pancreatic head. The patient was diagnosed with resectable pancreatic head cancer without lymph node metastasis (cT1cN0M0, cStageIA) according to the Union for International Cancer Control, 8th edition. As a standard pancreatic cancer treatment, two courses of preoperative chemotherapy with gemcitabine plus S-1 were administered. CT after preoperative chemotherapy identified no significant changes in tumor size but revealed SMA stenosis due to atherosclerosis. Blood flow to the left-sided colon was supplied from the middle colic artery via the SMA because of the past sigmoidectomy with inferior mesenteric artery detachment. Therefore, SMA stent placement was performed 1 day preoperatively. Subsequently, pylorus-preserving pancreatoduodenectomy (PPPD) was performed, preserving the remnant stomach with the right gastroepiploic (RGE) artery and vein. After resection, indocyanine green fluorescence imaging confirmed a good blood supply to the remnant stomach. The operation time was 467 min, and the blood lost was 442 mL. Histopathologically, the tumor was diagnosed as moderate adenocarcinoma and pT1cN0M0, Stage IA. The postoperative course was uneventful. The patient was discharged on postoperative day 23. S-1 as adjuvant chemotherapy was administered on postoperative day 63. The patient has been alive without recurrence for 7 months.

**Conclusions:**

We performed PPPD preserving blood supply for pancreatic head cancer in a patient with benign SMA stenosis and a history of PG and sigmoidectomy. Blood supply was preserved through preoperative SMA stent placement and a surgical procedure preserving the RGE vessels. Furthermore, S-1 adjuvant chemotherapy was successfully initiated. These multimodal therapies contributed to a favorable clinical outcome.

## Background

With an increasing elderly population, there is a growing incidence of performing pancreatoduodenectomies in patients with a history of abdominal surgery and systemic atherosclerosis. Particularly, blood supply to the remnant stomach should be preserved when performing a pancreatectomy in patients with a history of gastrectomy. Moreover, ischemic complications should be considered when performing pancreatoduodenectomy (PD) in patients with celiac axis (CA) and superior mesenteric artery (SMA) stenosis. However, whether these surgical procedures can be safely performed remains unclear. Herein, we described a case of pylorus-preserving PD (PPPD) preserving blood supply for pancreatic cancer in a patient with SMA stenosis and a history of proximal gastrectomy (PG) and sigmoidectomy.

## Case presentation

**Fig. 1:**
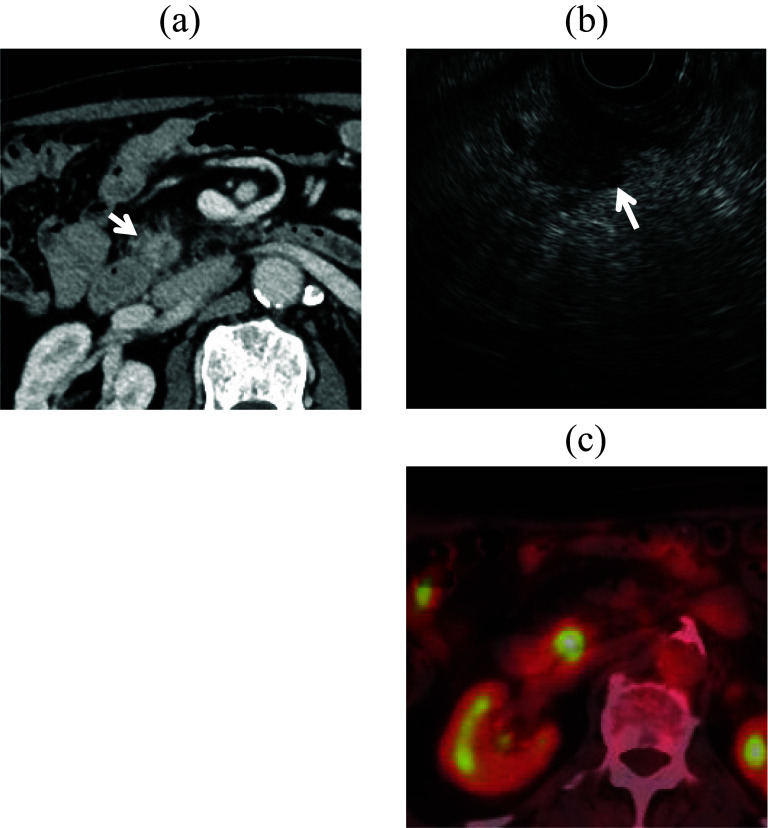
**a** Computed tomography (CT) shows an irregular nodular tumor of 13 mm in the pancreatic head without superior mesenteric vein and artery involvement. **b** Endoscopic ultrasonography shows a hypoechoic tumor. **c** 18F-fluorodeoxyglucose positron emission tomography–CT (FDG PET–CT) shows high FDG accumulation in the nodular tumor without high FDG accumulation in other organs

**Fig. 2:**
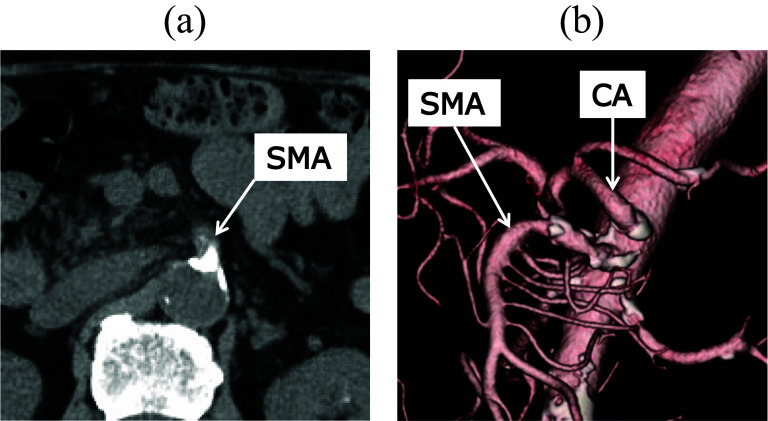
**a** Computed tomography (CT) shows superior mesenteric artery (SMA) stenosis due to atherosclerosis. **b** Three-dimensional image reconstructed from dynamic CT also shows SMA stenosis

**Fig. 3:**
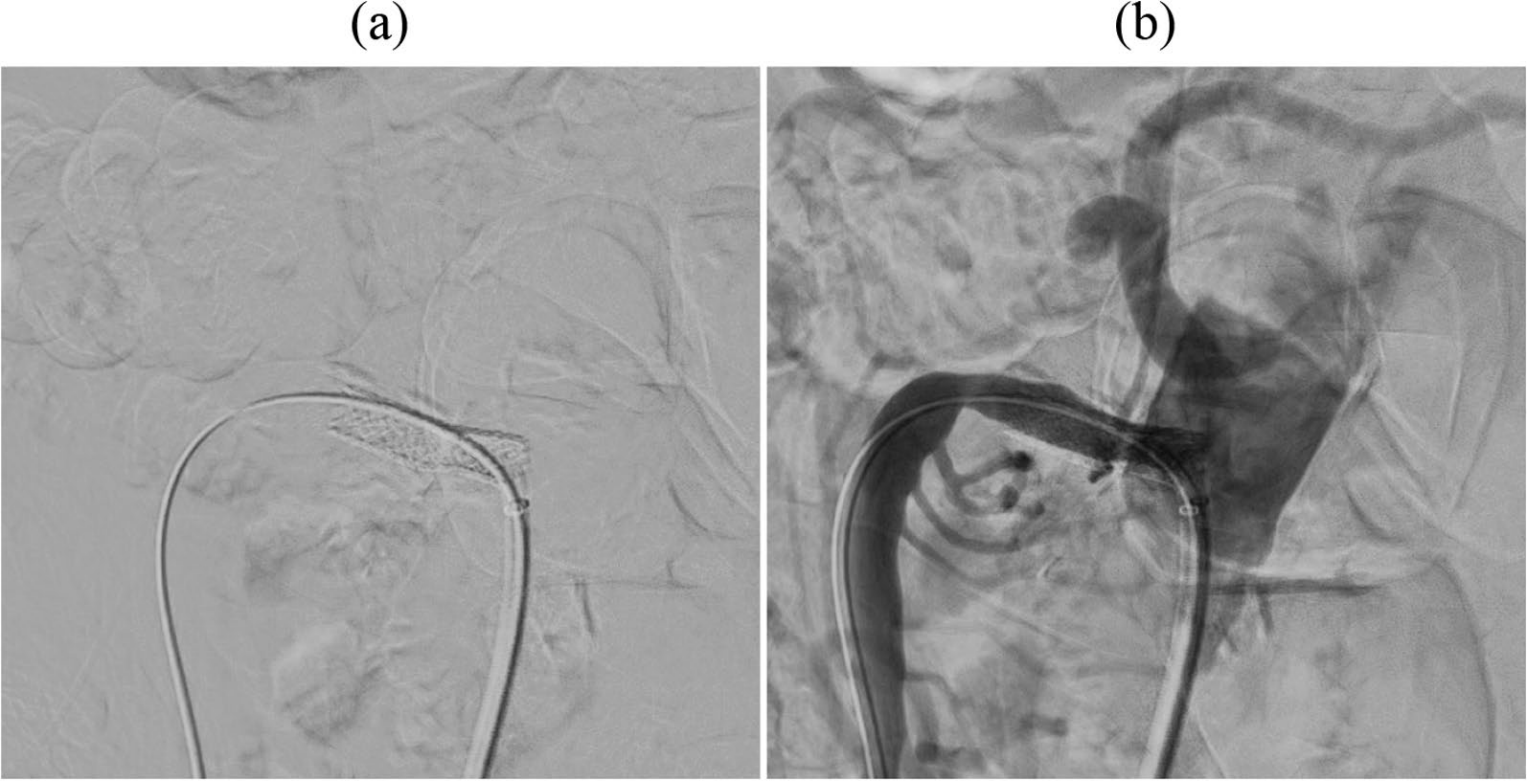
**a, b** Angiography showed stenosis at the root of the superior mesenteric artery and post-stenotic dilatation. Endovascular stent placement performed one day preoperatively

A 75-year-old man had a history of laparoscopic PG with double-flap technique for early gastric cancer and laparoscopic sigmoidectomy for advanced sigmoid cancer 4 years previously. Follow-up computed tomography (CT) revealed an irregular nodular tumor measuring 13 mm in the pancreatic head (Fig. 1a). The tumor did not involve major vessels such as the gastroduodenal artery (GDA), common hepatic artery, CA, SMA, or superior mesenteric vein. Biliary stricture or dilatation of the main pancreatic duct was not observed. The patient did not have jaundice. Blood test results showed that serum total bilirubin, aspartate aminotransferase, alanine aminotransferase, carcinoembryonic antigen, and carbohydrate antigen 19-9 levels were within reference ranges. Endoscopic ultrasonography showed a hypoechoic tumor (Fig. 1b). Positron emission tomography CT showed high fluorodeoxyglucose (FDG) accumulation in the nodular tumor without high FDG accumulation in other organs (Fig. 1c). Therefore, the patient was diagnosed with a resectable pancreatic head cancer without lymph node metastasis and vascular involvement (cT1cN0M0, cStageIA) according to the Union for International Cancer Control (UICC), 8th edition. As a standard pancreatic cancer treatment, two courses of preoperative chemotherapy with gemcitabine plus S-1 (gemcitabine 1000 mg/m^2^ on days 1 and 8, plus S-1 100 mg daily on days 1–14 of a 21-day cycle) were administered. CT after preoperative chemotherapy identified no significant change in tumor size but revealed SMA stenosis due to atherosclerosis (Fig. 2a, b). Blood flow of the left-sided colon was supplied from the middle colic artery (MCA) via the SMA owing to the past sigmoidectomy with inferior mesenteric artery (IMA) detachment. During the postoperative period, SMA occlusion can lead to extensive small bowel and right-sided colon necrosis, as well as left-sided colon necrosis due to interruption of the blood supply from the MCA, which can be fatal. Therefore, SMA stent placement was performed 1 day preoperatively (Fig. 3a, b). After SMA stent placement, heparin was administered intravenously up to 6 h before the start of surgery. Subsequently, PPPD preserving the right gastroepiploic vessels was performed.

**Fig. 4:**
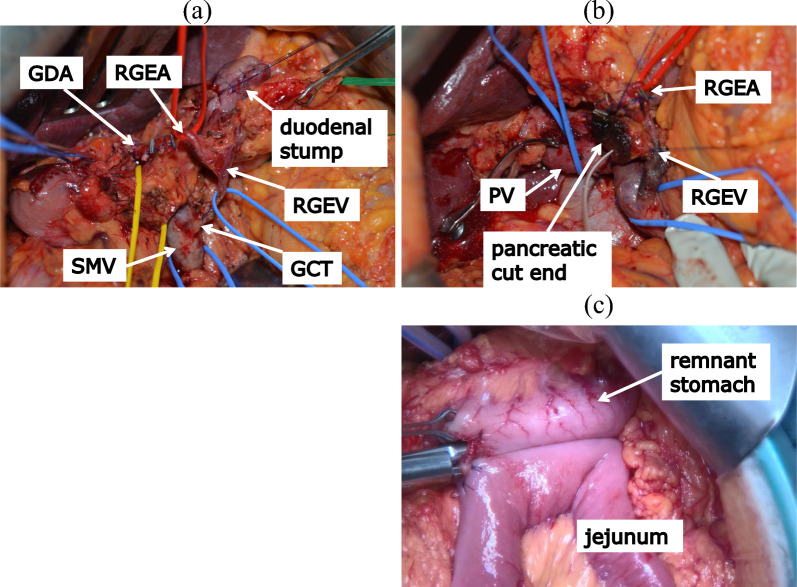
Intraoperative findings. The right gastroepiploic artery and vein are preserved via the gastroduodenal artery and gastrocolic trunk, respectively. **a** After isolating the right epiploic vessels from pancreas. **b** After pylorus-preserving pancreatoduodenectomy. **c** Arterial ischemia and venous congestion are not found in the remnant stomach. Gastrojejunostomy between the posterior wall of the remnant stomach and jejunum is performed using side-to-side anastomosis via the antecolic route

**Fig. 5:**
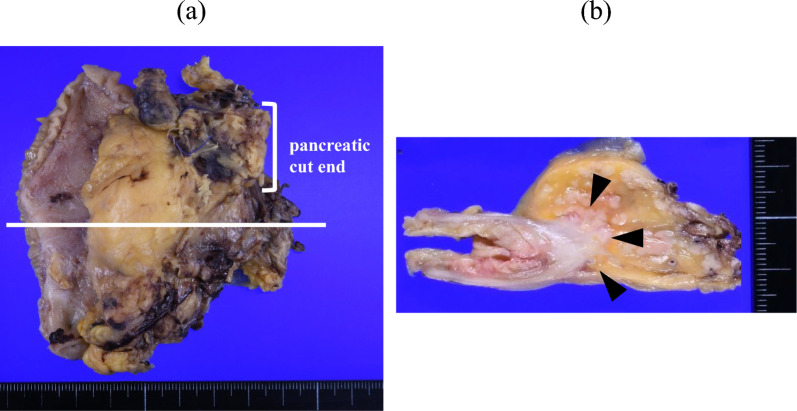
**a, b** Macroscopic findings of the resected specimen show an irregular nodular tumor in the pancreatic head (arrow head). (**b**, cross section at line)

A midline incision was made. Adhesiolysis and intraabdominal exploration were performed. No liver and peritoneal metastases were observed. PPPD was performed, preserving the right gastroepiploic artery (RGEA) via the GDA and right gastroepiploic vein via the gastrocolic trunk (GCT) (Fig. 4a, b). The right gastric artery and vein were divided. Arterial ischemia and venous congestion in the remnant stomach were not observed macroscopically after resection. Moreover, indocyanine green (ICG) fluorescence imaging confirmed a good blood supply to the remnant stomach. A gastrojejunostomy between the posterior wall of the remnant stomach and jejunum was performed using a side-to-side anastomosis via the antecolic route (Fig. 4c). The operation time was 467 min, and blood loss was 442 mL. Histopathological examination revealed the surgical margin was negative for cancer (Fig. 5a, b). No lymph node metastasis or vascular invasion was observed. Finally, the tumor was diagnosed as moderate adenocarcinoma and pT1cN0M0, Stage IA according to UICC, 8th edition.

**Fig. 6:**
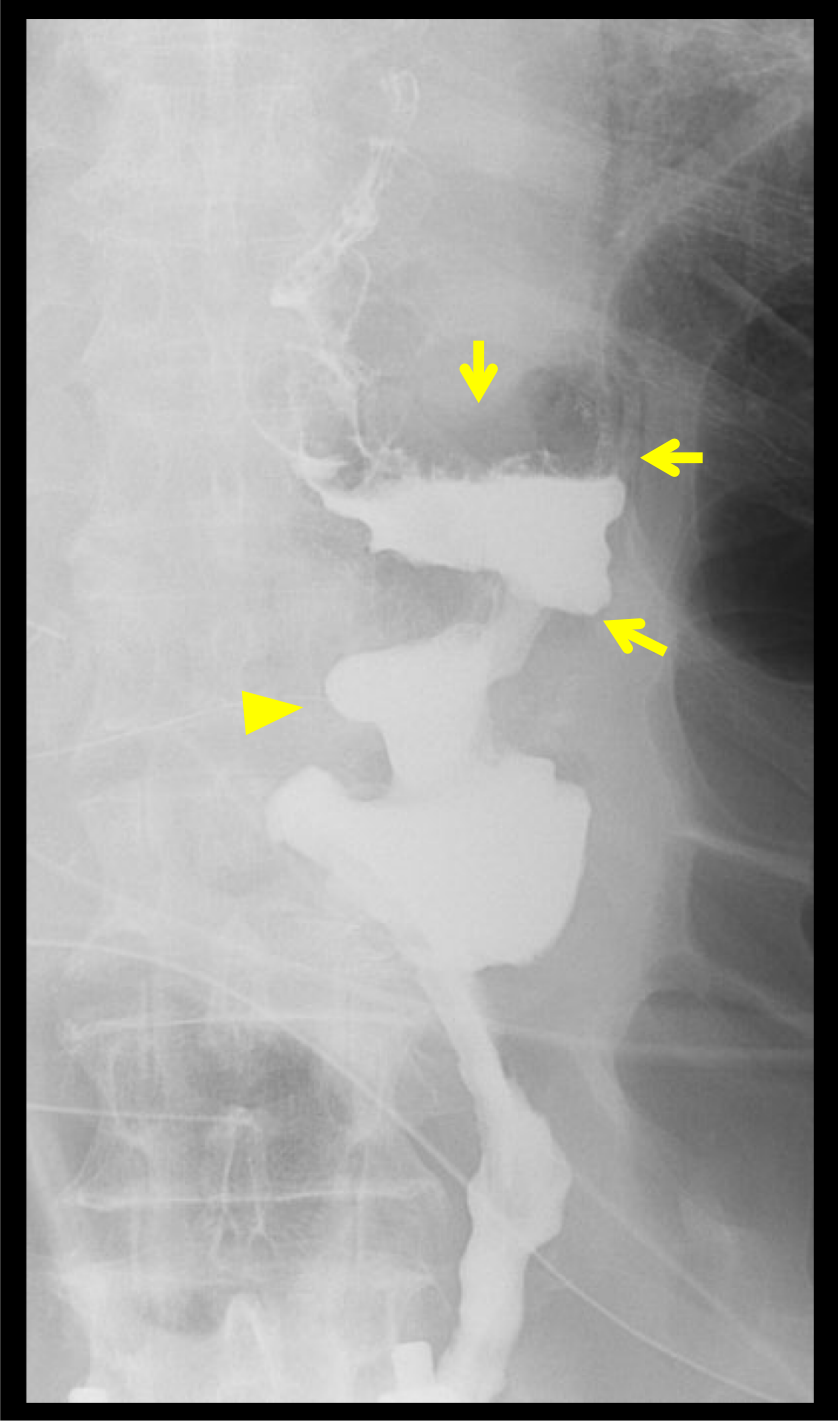
Postoperative gastric fluoroscopy with a water-soluble contrast medium. The contrast medium passes through the remnant stomach smoothly. The arrow and arrow head indicate the remnant stomach and gastrojejunostomy, respectively

The postoperative course was uneventful. Antithrombotic therapy for SMA stent was not administered postoperatively. Postoperative gastric fluoroscopy with water-soluble contrast medium showed smooth flow from the esophagus to the jejunum (Fig. 6). The patient was discharged on postoperative day 23.

S-1 as adjuvant chemotherapy was administered on postoperative day 63. Although the patient lost 7 kg compared with his preoperative body weight, the amount of oral intake remained unchanged compared with preoperative values. The patient recovered from normal serum albumin, total protein, and cholinesterase levels to within normal ranges 4 months postoperatively. The patient has been alive without recurrence for 8 months.

## Discussion

Herein, we described a case of PPPD preserving blood supply for pancreatic cancer in a patient with SMA stenosis and a history of PG and sigmoidectomy. We achieved both radical resection and preservation of organ function using multimodal treatment combined with endovascular stenting and surgical resection preserving blood flow in the remnant stomach.

The preservation of blood supply to the remnant stomach should be considered when performing pancreatectomy after gastrectomy [1]. In a typical PG, the left gastric artery, left gastroepiploic artery, and short gastric artery are divided. Therefore, blood flow to the remnant stomach is supplied by the right gastric artery and RGEA. When performing PD after PG, three surgical procedures can be considered: (1) preservation of the GDA–RGEA and the remnant stomach [2, 3], (2) division and reconstruction of the GDA and RGEA and preservation of the remnant stomach [4], and (3) removal of the GDA and remnant stomach and reconstruction of the alimentary tract with the small intestine. When removing the GDA and remnant stomach after PG, remnant gastrectomy using left thoracotomy must be performed in addition to the PD. However, the method is invasive. Loss of both the stomach and pancreas can lead to severe postoperative nutritional imbalance and derangement of glucose metabolism [5]. Ideally, maximum organ function should be preserved while achieving R-0 resection. The significance of lymph node dissection (LND) around the pyloric ring on long-term outcome remains unclear, and there were no studies on whether preservation of the GDA–RGEA can lead to insufficient LND [6]. Thus, surgical procedures (1) or (2) should be selected according to tumor involvement and the presence or absence of lymph node metastasis. There have been two cases in which PD involved removal of the GDA–RGEA or postoperative coil embolization for a pseudoaneurysm of the GDA while preserving the remnant stomach [7, 8]. However, in order to reliably and safely preserve the remnant stomach, it is preferred to preserve or reconstruct the GDA–RGEA. In our case, preoperative CT revealed that the tumor did not infiltrate GDA–RGEA, and no lymph node metastasis was observed. Therefore, PPPD preserving the GDA–RGEA and remnant stomach was selected. Histopathological examination of the resected specimen revealed an R-0 resection with no lymph node metastases or vascular invasion.

Extended LND does not provide survival benefits [9–13]. Nagakawa et al. reported that the survival benefits of regional LND in pancreatic cancer remain unclear and that performing LND to achieve R0 resection is necessary for long-term survival [14]. In addition, preoperative and postoperative chemotherapy are essential for improving the prognosis of pancreatic cancer [15–17]. The survival benefit of early initiation of S-1 adjuvant chemotherapy has also been reported [18]. Hence, in pancreatic cancer treatment, surgery aimed at R0-resection, while preserving organ function, is necessary, followed promptly by adjuvant chemotherapy with S-1.

In our case, RGEV–GCT was also preserved. Insufficient blood flow leads to anastomotic leakage. Improving the circulation of the anastomotic site can reduce the incidence of anastomotic leakage [19]. Preserving both venous return and arterial input flow is important for avoiding congestion and ischemia. Indeed, in the supercharge technique for esophageal and pharyngeal reconstruction to augment blood flow, both the arteries and veins are anastomosed to the recipient vessels [20]. However, PD for pancreatic cancer is often combined with portal vein resection. RGEV reconstruction should be considered when RGEV–GCT cannot be preserved [21].

Recently, the usefulness of Doppler ultrasonography, tissue oxygen saturation, and ICG fluorescence has been reported as methods for evaluating the blood supply in the digestive tract [4, 22, 23]. ICG fluorescence has been widely used to assess blood flow during gastrointestinal reconstruction, sentinel lymph node mapping, liver tumor identification, hepatic segment staining during liver resection, and cholangiography during cholecystectomy. In our case, we confirmed blood supply to the remnant stomach using ICG fluorescence. The stomach functioned well postoperatively. Although the patient lost body weight, the amount of oral intake remained unchanged, and serum albumin, total protein, and cholinesterase levels, used as nutritional markers, improved to within the normal range.

In the present case, an SMA stent was placed preoperatively to treat benign SMA stenosis due to atherosclerosis. Benign SMA stenosis is often caused by atherosclerosis or isolated SMA dissection, and stent placement is indicated only in symptomatic cases [24]. Endovascular revascularization using SMA stent is relatively less invasive and safer compared to open surgical approach, with lower rates of restenosis and reintervention [25]. However, there are no studies on the efficacy of SMA stenting in patients undergoing PD with SMA stenosis. To the best of our knowledge, only two reports have mentioned pancreatectomy after SMA stent placement for benign SMA stenosis [26, 27]. In PD, the pancreaticoduodenal arcade is divided. Therefore, even if the SMA occludes after PD, it will not be able to receive blood supply from the CA. In addition, in our case, blood flow could not be supplied from the MCA–IMA arcade due to a previous sigmoidectomy with IMA detachment. Patients with both SMA and IMA stenosis have a higher risk of developing mesenteric ischemia than patients with isolated SMA stenosis [28]. Postoperatively, SMA occlusion could lead to not only extensive small bowel and right-sided colon necrosis but also left-sided colon necrosis due to interruption of the blood supply from the MCA. To prevent this life-threatening complication, an SMA stent was placed preoperatively, which resulted in a favorable postoperative course. Therefore, we suggest preoperative SMA stent placement when performing PD for patients with both SMA and IMA stenosis. If there was no indication for surgery, preservation of the pancreaticoduodenal arcade suggests that SMA stenting may not be warranted.

## Conclusion

We performed PPPD preserving blood supply for pancreatic head cancer in the patient with benign SMA stenosis and a history of PG and sigmoidectomy. Blood supply was preserved through preoperative SMA stent placement and a surgical procedure preserving the RGE vessels. Furthermore, S-1 adjuvant chemotherapy was successfully initiated. These multimodal therapies contributed to a favorable clinical outcome.

## Data Availability

The data are not available for public access because of patient privacy concerns but are available from the corresponding author on reasonable request.

## Abbreviations

CA: Celiac axis
FDG: Fluorodeoxyglucose
GDA: Gastroduodenal artery
GDA–RGEA: Gastroduodenal artery–right gastroepiploic artery
IMA: Inferior mesenteric artery
MCA: Middle colic artery
PD: Pancreatoduodenectomy
PG: Proximal gastrectomy
PPPD: Pylorus-preserving pancreatoduodenectomy
RGEA: Right gastroepiploic artery
SMA: Superior mesenteric artery
